# Glioma facilitates the epileptic and tumor-suppressive gene expressions in the surrounding region

**DOI:** 10.1038/s41598-022-10753-4

**Published:** 2022-04-26

**Authors:** Kazuki Komiyama, Keiya Iijima, Reika Kawabata-Iwakawa, Kazuyuki Fujihara, Toshikazu Kakizaki, Yuchio Yanagawa, Yuhei Yoshimoto, Shigeo Miyata

**Affiliations:** 1 Department of Neurosurgery, Gunma University Graduate School of Medicine, Maebashi, Japan; 2 Department of Neurosurgery, National Center Hospital, National Center of Neurology and Psychiatry, Kodaira, Japan; 3 Division of Integrated Oncology Research, Gunma University Initiative for Advanced Research, Maebashi, Japan; 4 Departments of Psychiatry and Neuroscience, Gunma University Graduate School of Medicine, Maebashi, Japan; 5 Department of Genetic and Behavioral Neuroscience, Gunma University Graduate School of Medicine, Maebashi, Japan

**Keywords:** Cancer, Neuroscience

## Abstract

Patients with glioma often demonstrate epilepsy. We previously found burst discharges in the peritumoral area in patients with malignant brain tumors during biopsy. Therefore, we hypothesized that the peritumoral area may possess an epileptic focus and that biological alterations in the peritumoral area may cause epileptic symptoms in patients with glioma. To test our hypothesis, we developed a rat model of glioma and characterized it at the cellular and molecular levels. We first labeled rat C6 glioma cells with tdTomato, a red fluorescent protein (C6-tdTomato), and implanted them into the somatosensory cortex of VGAT-Venus rats, which specifically expressed Venus, a yellow fluorescent protein in GABAergic neurons. We observed that the density of GABAergic neurons was significantly decreased in the peritumoral area of rats with glioma compared with the contralateral healthy side. By using a combination technique of laser capture microdissection and RNA sequencing (LCM-seq) of paraformaldehyde-fixed brain sections, we demonstrated that 19 genes were differentially expressed in the peritumoral area and that five of them were associated with epilepsy and neurodevelopmental disorders. In addition, the canonical pathways actively altered in the peritumoral area were predicted to cause a reduction in GABAergic neurons. These results suggest that biological alterations in the peritumoral area may be a cause of glioma-related epilepsy.

## Introduction

Glioma is the primary tumor occurring in the central nervous system. The median survival duration after initial diagnosis of glioblastoma (GBM, WHO grade 4) is 15 months^1,2^. Glioblastoma demonstrates extremely high potential for proliferation and aggressive invasion in the brain. The symptoms of increased intracranial pressure worsen rapidly. In addition, tumors usually invade deep into the brain, causing damage to important brain functions, which can be fatal. Invasion of the motor nerves can easily result in paralysis. Surgical resection, chemotherapy, and radiation therapy are the standard treatments for GBM. However, due to highly aggressive invasion, complete removal is not possible, and even with multidisciplinary treatment, many patients do not survive for 1 year. The chemotherapeutic agent temozolomide increases the median survival duration in patients with GBM; however, it does not lead to dramatic improvements in life outcome^2,3^.

Approximately 60–80% of patients with glioma and 23–50% of patients with GBM develop epilepsy^4–7^. The primary symptom of epilepsy in patients with malignant brain tumors is seizures accompanied by sudden limb spasms and loss of consciousness, leading to motor and cognitive dysfunction such as Todd's palsy. In an animal model of brain tumors, abnormal neural firings (burst discharges) have been observed in the peritumoral area^8^. We recently reported that burst discharges were also found in the peritumoral area in patients with malignant brain tumors during biopsy^9^. Therefore, burst discharges in the peritumoral area may be an epileptic focus in patients with malignant brain tumors.

Several nonclinical studies have suggested that the excitation/inhibition imbalance elicits burst discharges in the peritumoral area^10,11^. The expression of cystine glutamate transporter system xc (xCT) was upregulated on the surface of glioma cells, and the excitatory amino acid glutamate was released from glioma mediated by xCT. In addition, the function of excitatory amino acid transporter 1 (EAAT1) and EAAT2, which take up synaptic glutamate, was impaired in astrocytes surrounding brain tumors, leading to the accumulation of glutamate in the extracellular space^10^. The increase in extracellular glutamate activates AMPA and NMDA receptors, triggering an influx of Na^2+^ and Ca^2+^ into neurons, and may cause burst discharges. On the other hand, the number of parvalbumin-positive GABAergic neurons was significantly reduced in the peritumoral area in a mouse model of glioma, indicating that a decrease in inhibitory neurotransmission disrupts the excitation/inhibition balance, leading to neural circuit hyperexcitability and tumor-related epilepsy^11,12^. It was recently reported that glioma-derived thrombospondin-2 (TSP2) promotes excitatory synapse formation and results in hyperexcitability in the peritumoral region^13^. This means that the glioma-neuron interaction in the peritumoral area is crucial for developing epileptic discharges. Based on these reports, the microenvironment surrounding brain tumors may be biologically altered. It is important to examine the biological alterations in the peritumoral area to resolve the pathophysiology of epilepsy accompanied by brain tumors.

High throughput omics techniques have facilitated the understanding of biological alterations corresponding to diseases specific to organs, tissues, and cells. RNA sequencing (RNA-seq) followed by pathway analyses is a powerful tool to resolve biological alterations in disease areas. The data of differentially expressed genes (DEGs) in the peritumoral area will be beneficial to understand the pathophysiology of epilepsy accompanied by brain tumors. To perform RNA-seq, selective dissections of peritumoral tissues are necessary, and the laser capture microdissection (LCM) system can be applied for this purpose. In this study, we performed transcriptomic analysis in the peritumoral area in a rat model of glioma by using a combination of LCM and RNA-seq (LCM-seq) to resolve the pathophysiology of glioma-related epilepsy.

## Results

### Development of VGAT-Venus rat glioma model implanted with C6-tdTomato cells

**Figure 1:**
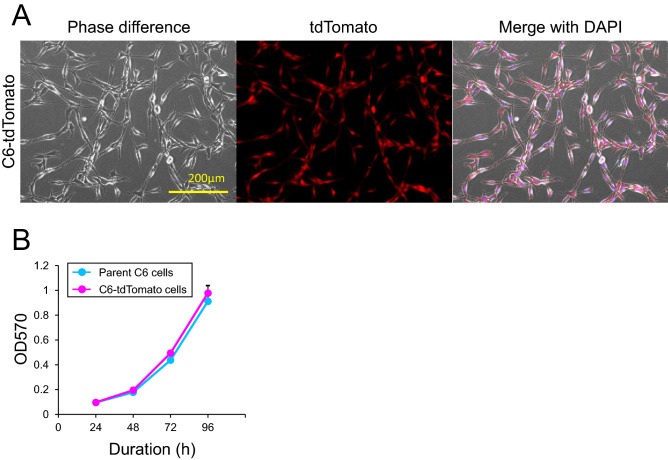
Characterization of C6-tdTomato cells. (**A**) Phase-contrast and fluorescence microscopy images of C6-tdTomato cells. (**B**) Viability of C6-tdTomato cells and their parent C6 glioma cells. Cell viability was assessed by the MTT assay and repeated four times. The averaged values of OD570 with SE are demonstrated as a line graph.

The rat C6 glioma cells is well used as an experimental model for the study of GBM growth and invasion^14^. The implantation of C6 glioma cells into rat brains elicits epileptic neural firings in the peritumoral region^13^. Therefore, in this study, we chose this model for investigating the pathophysiology of glioma-related epilepsy. To easily identify the peritumoral area and GABAergic neurons, we developed a VGAT-Venus transgenic rat glioma model implanted with tdTomato-labeled C6 cells. Rat C6 glioma cells were labeled with tdTomato, a red fluorescent protein by stable transfection with tdTomato and PGK-neo plasmids, and the resultant cells were referred to as C6-tdTomato cells (Fig. 1A). The morphology and cell viability of C6-tdTomato cells were comparable to those of the parent C6 glioma cells (Fig. 1B).

**Figure 2:**
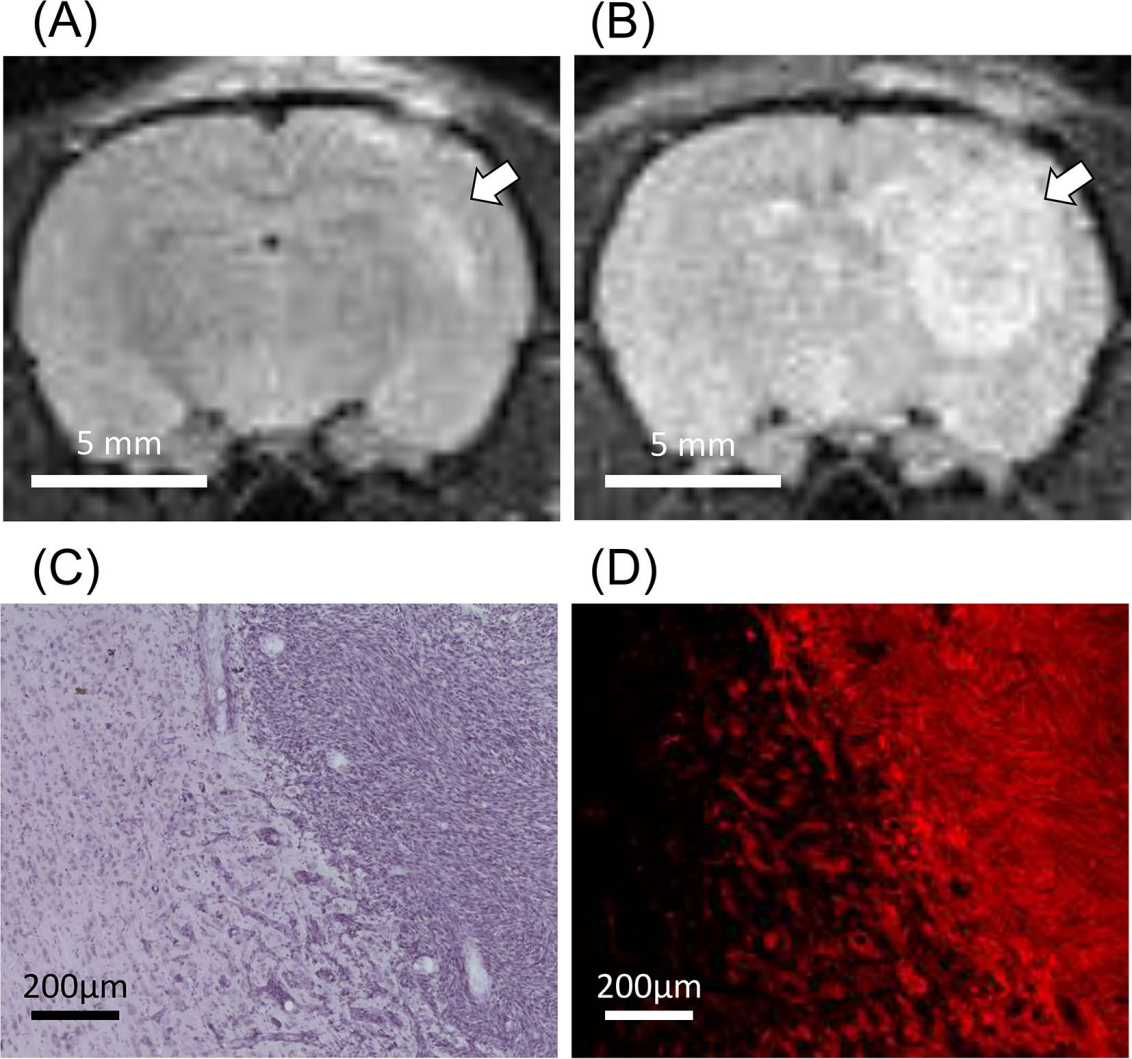
Development of the rat glioma model by implantation of C6-tdTomato cells into the brain. (**A**,**B**) T2-weighted MR images of the rat brain implanted with C6-tdTomato cells at 4 days and 10 days after the surgery. The tumor area was indicated by a white arrow. (**C**,**D**) Serial sections of C6-tdTomato-implanted rat brains were made 10 days after surgery, one section was stained by Hematoxylin–Eosin staining, and another section was observed by the fluorescence microscopy.

C6-tdTomato cells (10^6^ cells in 1 µL PBS) were implanted into the somatosensory cortex of male Wistar rats. Four days after implantation, tumor growth was measured by MRI, and the size of the tumor was monitored everyday (Fig. 2A,B). Nine to 14 days after implantation, the brain was fixed with 4% paraformaldehyde (PFA), and then serial 20 μm sections of the brains were prepared. Tumor localization and its approximate size could be determined by Hematoxylin–Eosin (HE) staining with bright field microscopy. However, tumor edges and glioma cells infiltrating into brain tissue were hard to identify by HE staining (Fig. 2C). On the other hand, the tumor edges and infiltrated glioma cells were easily identified with fluorescence microscopy in C6-tdTomato-implanted brain sections (Fig. 2D).

**Figure 3:**
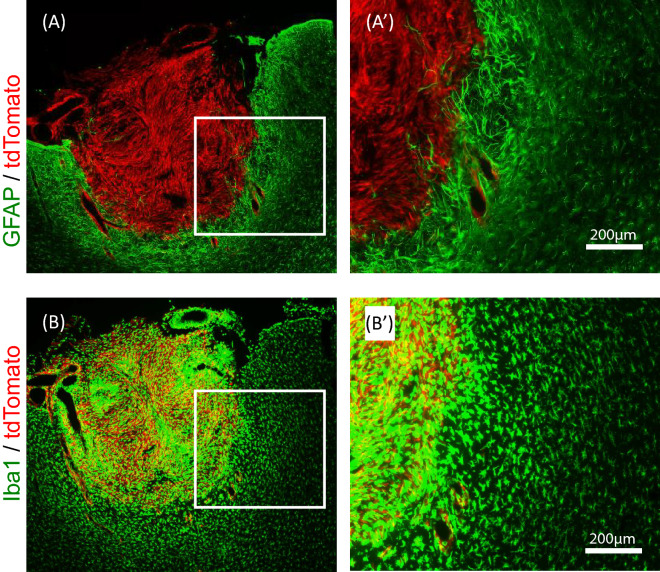
Localization of astrocytes and microglia in the peritumoral area of C6-tdTomato implanted rats. (**A**,**A**′) Astrocytes around tumors demonstrate activated forms and highly express GFAP. (**B**,**B**′) Microglia around tumors demonstrate activated forms and highly express Iba1.

It has been reported that astrogliosis occurs at the tumor margin and adjacent tissue, forming a glial scar^15^. Accordingly, we observed that GFAP immunoreactivity was highly upregulated in astrocytes localized at tumor margins, and that GFAP-upregulated astrocytes exhibited a reactive structure (Fig. 3A,A′). We also examined the localization and structure of microglia by staining for Iba1. Iba1-positive (but tdTomato-negative) cells accumulated at tumor margins, and these cells largely exhibited the reactive form (Fig. 3B,B′). Iba1-positive cells were also observed in the tumor core; however, we could not distinguish whether these cells were host microglia or C6-tdTomato cells because the Iba1 protein is expressed in both microglia and C6 glioma cells.

### Density of GABAergic inhibitory neurons in the peritumoral area

**Figure 4:**
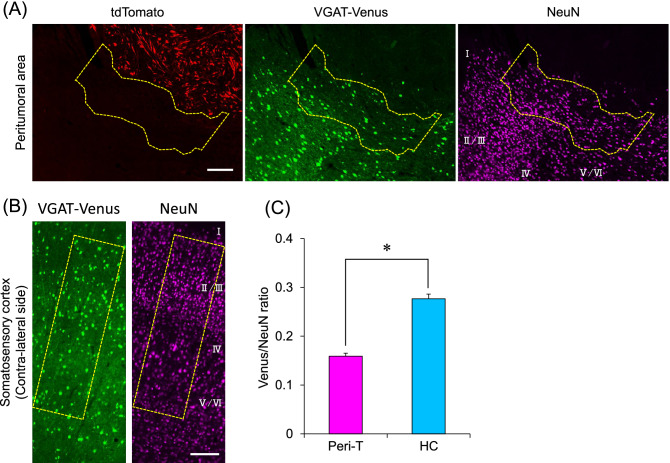
Densities of excitatory and inhibitory neurons in the peritumoral area. Representative images showing the cells labeled with tdTomato, Venus, and NeuN in the peritumoral area (**A**) and in the contralateral side (**B**) of the somatosensory cortex of the same rat. The number of Venus-positive and NeuN-positive neurons was counted in layers II to VI within the dotted line and the ratio of Venus-positive/NeuN-positive neurons was calculated (**C**). The data were obtained from five sections from five rats (total 25 sections). The means with SE are demonstrated as column. *P < 0.05 between healthy control (HC) and peritumoral tissues (Peri-T) (Student’s t-test). The white bars indicate 200 μm.

Next, we determined the imbalance of excitatory/inhibitory neurons in the peritumoral area. To easily count the number of GABAergic neurons, VGAT-Venus-B rats^16^ were used for the experiments. The number of Venus-positive GABAergic neurons and NeuN-positive neurons were counted in the peritumoral region, which was defined as the area up to 400 µm away from the tumor edges (Fig. 4A). The number of Venus-positive and NeuN-positive cells was also counted on the contralateral side of the somatosensory cortex of the same rats (Fig. 4B). The ratio of Venus-positive/NeuN-positive cells was significantly decreased in the peritumoral regions compared with the contralateral healthy regions (Fig. 4C). These results indicate that the density of GABAergic inhibitory neurons is decreased in the peritumoral area.

### Transcriptomic analysis in the peritumoral area

**Figure 5:**
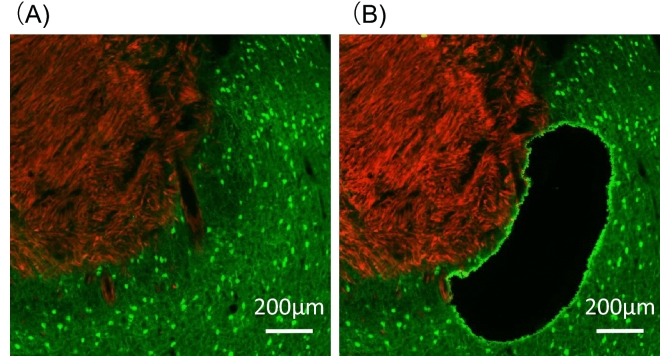
Representative images of brain sections before (**A**) and after (**B**) LCM.

Similar to the findings of the present experiments and previous studies^15,17^, the microenvironment surrounding brain tumors may be biologically altered, but the details have not yet been investigated. To resolve this question, we aimed to perform transcriptomic analysis in peritumoral tissues (Peri-T). We first dissected the Peri-T from unfixed brain sections by LCM with a fluorescence microscope, but it was hard to discern the peritumoral area in these sections. To overcome this problem, we fixed the brain sections by PFA. The peritumoral area of PFA-fixed brain sections was easily discerned and could be dissected by LCM equipped with a fluorescence microscope (Fig. 5). However, it is well known that RNA integrity in tissues is degraded by formalin fixtation^18^. Therefore, we used a RNA-seq kit adequate for degraded RNA obtained from formalin-fixed paraffin-embedded (FFPE) tissues.

**Figure 6:**
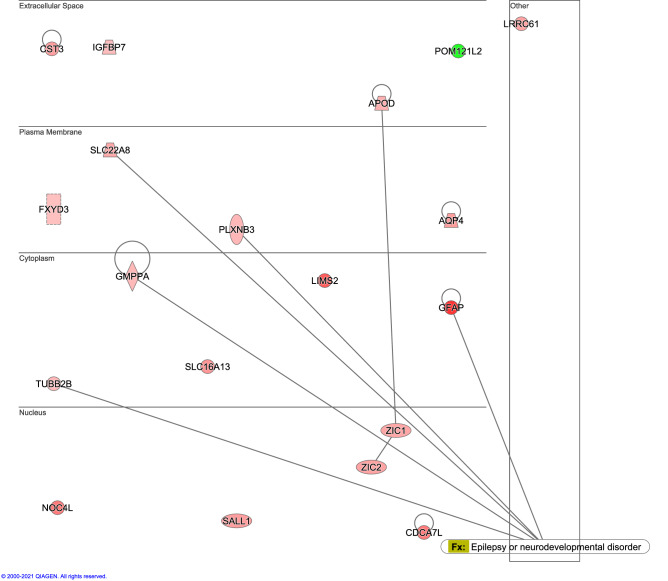
Association image of 19 differentially expressed genes (DEGs) with diseases and functions. Ingenuity Pathway Analysis revealed that the 5 DEGs were associated with epilepsy and neurodevelopmental disorders.

**Figure 7:**
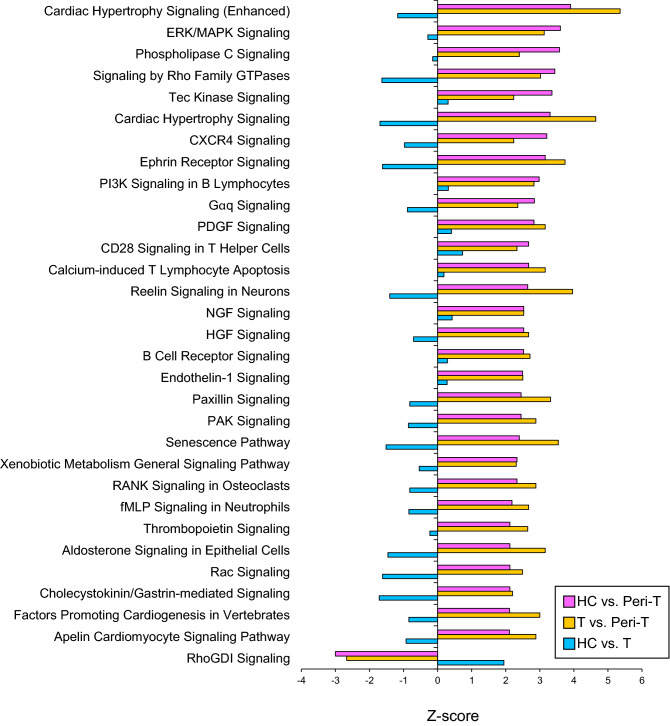
Canonical pathways predicted to be actively altered in the peritumoral area. The results of canonical pathways between two groups were used as the dataset for the comparison analysis. Z-scores greater than 2 (or lower than -2) were considered significant. Significant canonical pathways specific to peritumoral tissues (Peri-T) are shown in the bar graph.

By comparing the genes expressed in the healthy (HC) and tumor (T) tissues, a total of 19 genes were differentially expressed in the Peri-T (Table 1). Among them, 18 genes were upregulated, and one gene (*Pom121l2*) was downregulated. The association of the 19 DEGs with “diseases and functions” was examined in the IPA database. Among them, IPA revealed that 5 genes (*Gfap, Gmppa, Tubb2b, SLC22a8* and *Plxnb3*) were related to epilepsy or neurodevelopmental disorders (Fig. 6). The number (5 of 19 genes) of matches to the disease and function annotation in the IPA database was significantly higher than the theoretical number of matches (p < 0.001). Next, we performed a comparison analysis in the IPA to identify other biological alterations in the Peri-T. The 31 canonical pathways predicted to be actively altered in the Peri-T group compared with the HC and T groups are shown in Fig. 7.

**Table 1:** DEG list.

Gene symbol	Peri-T/HC		Peri-T/T		T/HC	
	log2(FC)	q-values	log2(FC)	q-values	log2(FC)	q-values
Gfap	3.71	4.65E−23	2.85	5.04E−10	0.75	0.372
Noc4l	2.26	3.93E−09	2.53	6.73E−11	− 0.42	0.649
Sall1	1.65	4.74E−05	1.62	7.00E−05	− 0.11	0.997
Cdca7l	2.27	1.16E−04	1.36	2.20E−02	0.83	0.599
Slc22a8	1.44	3.50E−04	1.70	5.71E−03	− 0.43	0.820
Lrrc61	1.56	4.65E−04	1.65	2.31E−04	− 0.21	0.888
Lims2	2.77	1.07E−03	3.42	8.57E−05	− 0.78	0.504
Zic2	1.57	1.16E−03	1.42	2.17E−03	0.00	1.000
Aqp4	1.67	1.59E−03	2.36	2.89E−06	− 0.84	0.183
Slc16a13	1.80	3.00E−03	2.13	7.28E−04	− 0.47	0.770
Tubb2b	1.20	5.83E−03	1.34	4.69E−03	− 0.22	0.904
Pom121l2	− 4.73	8.82E−03	− 5.20	7.21E−03	0.23	1.000
Zic1	1.49	9.23E−03	1.76	2.14E−02	− 0.41	0.883
Apod	1.32	1.14E−02	2.03	3.23E−05	− 0.83	0.295
Plxnb3	1.22	1.21E−02	1.73	1.52E−03	− 0.71	0.328
Cst3	1.65	2.16E−02	1.85	7.74E−03	− 0.26	0.951
Igfbp7	1.14	2.73E−02	1.17	1.31E−02	− 0.14	1.000
Gmppa	1.18	3.10E−02	1.51	3.13E−03	− 0.48	0.588
Fxyd3	1.07	4.70E−02	2.01	3.98E−05	− 1.05	0.177

Data obtained from LCM-seq were filtered to only include those results reaching q-values less than 0.05 between peritumoral tissues (Peri-T) and healthy tissues (HC) and between Peri-T and tumor (T), but not between HC and T. Genes are listed in order of decreasing q-values of Peri-T vs. HC. FC means fold change.

## Discussion

In this study, we developed a novel animal model of glioma by implanting C6-tdTomato cells into rat brains in which the tumor mass was visible even under a fluorescence microscope. By using this animal model, the discrimination of tumor localization and margins in brain sections was facilitated. In addition, we challenged the method of LCM-seq analysis from PFA-fixed tissues and found the upregulation of epilepsy-related genes in the peritumoral area. We also provided predictive canonical pathways in the peritumoral area, which will aid in understanding the pathophysiology of glioma-related epilepsy.

Reactive astrocytes were observed in the peritumoral area in the present model animals. Lee et al. (2011) previously reported that the monitoring of luciferase activity regulated by the GFAP promotor was useful to assess the temporal-spatial kinetics of host-mediated astrogliosis associated with glioma progression and metastatic brain tumor growth^15^. Instead of that model animal, we developed C6-tdTomato cells to discriminate the tumor localization and margins. Reactive astrocytes were assessed by immunofluorescent staining of the GFAP protein. As this combination, GFAP-positive astrocytes demonstrated an activating form around the tumor and allowed us to depict the association of reactive astrocytes with brain tumors more accurately.

Microglia are crucial immune cells in the brain and are activated by tissue damage and other factors. Glioblastoma development induces the activation of pro-inflammatory M1-polarized and anti-inflammatory M2-polarized microglia. It has been reported that peritumoral and tumor-infiltrating microglia can be activated into the M2-phenotype by various substances secreted by the tumor and contribute to tumor invasion, migration, and proliferation^19^. According to these reports, we observed reactive microglia at the tumor border and infiltrated the tumor mass.

In this study, we found that the density of GABAergic neurons was significantly decreased in the peritumoral area compared with the contralateral healthy area, suggesting that GABAergic neurons may be vulnerable in the microenvironment around the tumor. Similarly, a decreased number of parvalbumin-positive GABAergic neurons in the peritumoral area has been observed^12^. Studies in animal models of cerebral ischemia have demonstrated that GABAergic neurons are more vulnerable to hypoxic conditions than excitatory glutamatergic neurons^20,21^. Extensive hypoxemia may also be present surrounding glioblastoma. The expression of hypoxia-inducible factor (HIF-1) and vascular endothelial growth factor (VEGF) from tumor cells induces angiogenesis, but the hypoxic area has been shown to reside in areas away from blood vessels, including the peritumoral area^20^. In addition, GABAergic neurons are damaged under acidosis and alkalosis^22^. Various biological changes, such as hypoxia, occur in the peritumoral area, and cause a reduction in GABAergic neurons around the brain tumor.

To resolve the biological alterations in the microenvironment surrounding brain tumors, we performed LCM-seq. We identified that the expression levels of the 19 genes were significantly changed in Peri-T. Interestingly, 5 genes (*Gfap, Gmppa, Tubb2b, SLC22a8* and *Plxnb3*) among the DEGs are known to be related to epilepsy or neurodevelopmental disorders. Therefore, it is possible that the peritumoral area is an epileptic focus and these 5 genes may be involved in the pathophysiology of glioma-related epilepsy. Among the 19 DEGs, the top-ranked DEG was the astrocyte-marker Gfap, indicating that astrocytes were dense in the peritumoral area. In fact, we already demonstrated in the present immunohistochemistry assay that GFAP-positive astrocytes were enriched in the peritumoral area. Aqp4 (aquaporin 4) is highly expressed in astrocytes and regulates water homeostasis in the central nervous system^23,24^. Aqp4 is upregulated by the brain tumor edema^25^. Therefore, it is possible that the upregulation of the Gfap and Aqp4 genes was induced by astrogliosis and brain edema in the peritumoral area in C6-tdTomato-implanted rats. Several genes differentially expressed in Peri-T cells have a role in suppressing the progression of tumors and glioma cells. Sall1 (Spalt-Like Transcription Factor 1), which is expressed specifically in microglia and is associated with microglial activation, suppresses glioma cell proliferation and migration and may work as a functional tumor suppressor gene in glioma^26,27^. Zic1 (Zic family member 1) is associated with the suppression of glioma cell growth^28^. Plxnb3 (plexin-B3) inhibits cell migration and invasion induced by stimulation with its ligand Sema5A^29^. Apod (apolipoprotein D) might play a role in either decreased proliferation or cyst formation in pilocytic astrocytoma, ganglioglioma, subependymal giant cell astrocytoma, and pleomorphic xanthoastrocytomas^30^. Some genes differentially expressed in the peritumoral tissues contribute to the proliferation and infiltration of glioma cells. For example, Cdca7l promotes the proliferation and infiltration of glioma cells^31,32^. Based on these results, it is likely that host defense responses against glioma invasion are activated in the peritumoral area.

Next, we performed a comparison analysis and identified 31 canonical pathways with statistical significance in Peri-T. For example, ERK/MAPK signaling is associated with a variety of cellular functions, including metabolism, proliferation, division, motility, and apoptosis^33^. ERK signaling increases the proliferation and migration of glioma cells but the inhibition of ERK signaling increases the adhesion of glioma cells to the gelatin/collagen component of the ECM^34^. Phospholipase C signaling is also associated with cell proliferation as well as CXCR4^35^. Rac, RhoA, Cdc42 and other signaling pathways by Rho family GTPases are associated with glioma invasion^36^, and paxillin signaling is associated with migration and invasion^37^. It has been reported that PI3K and ERK/MAPK signaling mediate induction of HIF-1α^38^. Ephrin receptor signaling, which contributes to angiogenesis^39^, was also activated in the peritumoral area. Therefore, the peritumoral area is considered hypoxic. Because GABAergic neurons are vulnerable to hypoxia, the decreased density of GABAergic neurons in the peritumoral area may be elicited, at least in part, by the hypoxic state. The other pathways identified by IPA may also be altered in the peritumoral area, but further investigation is required to resolve their relevance to the pathophysiology of glioma-related epilepsy.

Non-progressive brain tumors, such as subependymal giant cell astrocytoma in tuberous sclerosis, is known to elicit the epileptic seizures with high rate (approximately 60–90% in the patients)^40^. It remains unclear whether the molecular mechanisms inducing epilepsy mediated by the non-progressive brain tumors are similar, or not, to those by progressive brain tumors. Further studies are needed to resolve this problem.

In conclusion, we suggest that biological alterations in the peritumoral area possibly cause glioma-related epilepsy.

## Methods

### Development of tdTomato-transfected C6 glioma cells

Rat C6 glioma cells (Cell number, JCRB9096; Lot number, 09112000) were purchased from Japanese Collection of Research Bioresources Cell Bank (National Institutes of Biomedical Innovation, Health and Nutrition). pBIKS(-)loxPPGKneopA (pPGKneo) conferring neomycin resistance, was kindly provided by Dr. J. Miyazaki at Osaka University. pCX-tdTomato containing tdTomato inserted into the pCAGGS expression vector was kindly provided by Dr. R. Kaneko at Osaka University.

C6 glioma cells were cultured in Dulbecco’s modified Eagle’s medium (D-MEM; FUJIFILM Wako Pure Chemical Corporation, Japan) supplemented with 10% fetal bovine serum (FBS; GIBCO, Thermo Fisher Scientific K.K., Japan), and 1% penicillin–streptomycin (FUJIFILM Wako Pure Chemical Corporation, Japan), and maintained under tissue culture conditions at 37 °C and 95% air, 5% CO_2_, and 100% humidity. Two micrograms of pPGKneo DNA and 3 µg of pCX-tdTomato DNA were mixed with Lipofectamine 3000 Reagent (Life Technologies, Thermo Fisher Scientific K.K., Japan) in Opti-MEM (Life Technologies) and applied to C6 glioma cells in a 60-mm culture dish according to the manufacturer’s protocol. Drug selection began 2 days later with the addition of 0.6 mg/mL G418 sulfate solution (FUJIFILM Wako) to the medium and was maintained for 13 days. Clonal G418-resistant cells were isolated by using cloning rings and expanded into 60-mm dishes. Then, we confirmed that the G418-resistant cells expressed the tdTomato protein by fluorescence microscopy (BZ-X810, Keyence Co., Japan) and selected five clones, which demonstrated bright red fluorescence. Subsequently, we compared the morphology and viability of the five clones with those of parent C6 glioma cells and selected one clone demonstrating similar morphology and viability to the parent cells. We refer to this clone as C6-tdTomato.

Cell viability was evaluated by 3-(4,5-dimethyl-2-thiazolyl)-2,5-diphenyltetrazolium bromide (MTT; Tokyo Chemical Industry Co. Ltd., Tokyo, Japan) assay. In brief, the cells were seeded (1 × 10^3^ cells/well) in quintuplicate in four 96-well plates and cultured for 24, 48, 72 and 96 h in each plate. Then, the cells were treated with MTT solution (final concentration; 0.25 mg/mL) and further incubated under tissue culture conditions for 3 h. Afterward, the cells were lysed by 5% sodium decasulfonate (SDS; FUJIFILM Wako) in *N*,*N*-dimethylformamide (DMF; Nacalai Tesque, Inc., Kyoto, Japan) solution and the optical density at 570 nm (OD570) was measured by the microplate reader xMark (Bio-Rad Laboratories, Inc.) as the index of cell viability. The MTT assay was repeated four times, and the averaged values of OD570 with standard errors were calculated.

### Animals

All experiments were performed in accordance with the guidelines for the Animal Experimentation at Gunma University Graduate School of Medicine. All experimental protocols were evaluated and approved by the Gunma University Ethics Committee (permit number: 14-006, 19-009). This study complied with ARRIVE guidelines (https://arriveguidelines.org).

Male Wistar rats aged 8–9 weeks and male VGAT-Venus rats^16^ aged 9–13 weeks were used for the experiments. Wistar rats were purchased from CLEA Japan, Inc. (Tokyo, Japan) and acclimated to the laboratory environment several weeks before the experiments. VGAT-Venus rats were previously developed by us and have been housed in our laboratory. The animal room was maintained at 22 ± 3 °C with a 12-h light–dark cycle (lights on at 6:00, lights off at 18:00). The animals were housed with 2–3 rats per cage and had free access to food and water.

### Implantation of C6 glioma cells into rat brains

C6-tdTomato cells were implanted into the rat brains according to the implantation method^41^ with minor modifications. The somatosensory cortex (1.3 mm posterior and 4.5 mm lateral right to bregma, 3.5 mm depth) was targeted for implantation referenced to the rat brain atlas^42^.

Cultured C6-tdTomato cells were harvested, and then the cell suspension (1.0 × 10^6^ cells in 1 µL PBS) was prepared. Rats were anesthetized with continuous inhalation of isoflurane and placed in a stereotactic frame. After an incision was made in the scalp, a small hole was drilled into the skull. A 33-gauge needle (33G. Super Short, Dentsply Sirona, Japan) was inserted at the target area and the cell suspension (1 µL) was injected at a constant flow rate (1.0 µL/min). Two minutes after the injection, the needle was removed, and the incision was sutured. The glioma-implanted rats were then returned to the home cage.

### Magnetic resonance imaging (MRI)

Small animal MRI was carried out over time on days 4–14 after glioma implantation by a 1-T benchtop MR scanner (Icon; Bruker Biospin GmbH, Ettlingen, Germany) according to a previous report^9^. Anesthesia was induced by the inhalation of 5% isoflurane and maintained by the inhalation of 3% isoflurane in room air. The respiration rate was monitored throughout the procedure, and body temperature was maintained at 37 °C. The T2-RARE (Rapid Acquisition with Relaxation Enhancement) sequence was used to determine the colonization and growth of tumors. The measurement parameters were follows: rapid-acquisition relaxation enhancement factor 5, repetition time 2500 ms, echo time 60 ms with in-plane resolution of 30 × 30 mm^2^, thickness 1000 µm, and 5 slices.

### Immunofluorescence analysis

Animals were deeply anesthetized with continuous inhalation of isoflurane, and then fixed by perfusion with 4% paraformaldehyde (PFA) in 0.1 M PBS (pH 7.4) through the left ventricle. Thereafter, the brain was removed and postfixed overnight in 4% PFA at 4 °C.

Coronal sections (20 µm in thickness) were made by a vibrating blade tissue slicer (Neo-LinearSlicer MT, Dosaka EM Co., Ltd., Kyoto, Japan). After preincubation with 0.3% Triton X-100 and 2% BSA in PBS, the sections were incubated with primary antibodies in PBS containing 0.3% Triton X-100 and 2% BSA overnight at room temperature. These sections were rinsed in Tris-buffered saline with 0.1% Tween 20 and repeated three times. Thereafter, sections were incubated with secondary antibodies and DAPI (1:500, D523, Dojindo Laboratories, Japan) in PBS containing 0.3% Triton X-100 and 2% BSA for 30 min at room temperature. After rinsing, the stained sections were mounted on MAS-coated glass slides (Matsunami Glass Ind., Ltd., Osaka, Japan) with Fluoromount (K024, Diagnostic BioSystems, USA). Fluorescence images were captured with the fluorescence microscopy (BZ-X810, Keyence, Osaka, Japan).

The primary antibodies used in this study were mouse anti-neuronal nuclei (NeuN) (clone A60, 1:500, MAB377, Millipore Co.), rabbit anti-glial fibrillary acidic protein (GFAP) (1:100, GFAP-Rb-Af800, Frontier Institute Co. Ltd.) and rabbit anti-Iba1 (1:500, 019-19741, FUJIFILM Wako Pure Chemical Co.). In addition, the primary antibody of rabbit anti-green fluorescent protein (GFP) (1:1,000, GFP-Rb-Af2020, Frontier Institute Co. Ltd., Hokkaido, Japan) was used to enhance the fluorescent signal of Venus protein. The secondary antibodies used in this study were donkey anti-rabbit IgG conjugated with Alexa Fluor 488 (1:300, A-21206, Invitrogen) and donkey anti-mouse IgG conjugated with Alexa Fluor 647 (1:300, A-31571, Invitrogen).

Fluorescence images were captured with a fluorescence digital microscope (BZ-X810, Keyence, Osaka, Japan). The number of NeuN-positive cells and Venus-positive cells in the peritumoral area, and the contralateral healthy area was measured by ImageJ (v1.51) software (National Institutes of Health). Tewari et al. reported that the number of neurons (including parvalbumin-positive interneurons) was decreased within the area up to 600 µm from the tumor margin mediated by the implanted glioma cells^12^. Therefore, in this study we defined that the peritumoral area was the surrounding regions up to 400 µm from the tumor margin. The ratio of Venus-positive cells per NeuN-positive cell in each area was calculated. The data were obtained from five sections from five rats (total 25 sections).

Statistical analyses were conducted using BellCurve for Excel ver. 3.20 (Social Survey Research Information Co., Ltd., Tokyo, Japan). Significant differences between regions were assessed by Student’s t-test. Data are expressed as the mean with standard error (SE). Statistical significance was defined as a *p* value less than 0.05.

### Microdissection

For laser capture microdissection (LCM), the PFA-fixed brains of C6-tdTomato-implanted VGAT-Venus rats (n = 6) were prepared in accordance with the protocol described above. The brains were cut into 20 μm-thick coronal sections by a vibrating blade tissue slicer and mounted on 1 mm polyethylene naphthalate (PEN) membrane-coated slides (MembraneSlide NF 1.0 PEN, Order No. 415190-9081-000, Carl Zeiss Microscopy GmbH, Göttingen, Germany). We defined that the peritumoral area was the surrounding regions up to 400 µm from the tumor margin as mentioned above. The peritumoral tissues were microdissected by laser pressure catapulting using a Palm Zeiss UV laser capture microdissection system (PALM MicroBeam IV, ZEISS Version 4.6) equipped with a fluorescence microscope (ZEISS Axio Observer D1). The samples were collected into PALM AdhesiveCap 500 (Order No.415401-4400-255, Zeiss) according to the Carl Zeiss MicroImaging PALM protocol for RNA handling. Before and after microdissection, fluorescent images of brain slices were captured by the fluorescence microscopy (BZ-X810, Keyence, Osaka, Japan). The tissues of the tumor and the contralateral healthy area were manually dissected and collected.

### RNA extraction from dissected tissues

Total RNA was extracted from the dissected tissues by the High Pure FFPE RNA Micro Kit (Roche Diagnostics GmbH, Germany) according to the manufacturer’s instructions without performing the deparaffinization step. The RNA quantity and quality in the eluted samples were assessed using an Agilent Bioanalyzer (Agilent Technologies, Palo Alto, CA, USA) as recommended.

### RNA-seq

One-hundred fifty nanograms of total RNA was subjected to rRNA removal using the FastSelect-rRNA HMR Kit (Cat No.: 334386, QIAGEN Inc., Hilden, Germany), according to the manufacturer’s protocol. Library preparation was performed using the KAPA RNA HyperPrep Kit (Cat No.: KK8540, Nippon Genetics Inc., Tokyo, Japan) from rRNA removed RNA, according to the manufacturer’s protocol. The resulting libraries were subjected to paired-end sequencing using a NextSeq500 High Output v2.5 Kit (Cat No.: 2002490, Illumina Inc. San Diego, CA, USA) and the Illumina NextSeq 500 system (75-base paired-end reads; Illumina Inc.). Data processing and analyses were performed using STAR v2.5.2b^43^, samtools-1.2^44^ and HTSeq-0.6.1p1^45^. Briefly, reads were aligned against the UCSC Rattus norvegicus (Rat) reference genome 6 (rn6) from the iGenome webpage (https://support.illumina.com/sequencing/sequencing_software/igenome.html; Illumina Inc.) using a STAR pipeline with the default setting. Sorted BAM files by coordinate using samtools were subjected to generate a count matrix table by HTSeq with the -f bam -r pos -t exon option. Normalization and differentially expressed genes (DEGs) were detected with the TCC^46^ package of R version 3.6.2 (R Foundation for Statistical Computing, Vienna, Austria. https://www.R-project.org/). Genes with a false discovery rate (FDR)-adjusted p values less than 0.05 were defined as significantly DEGs.

### Ingenuity pathway analysis (IPA)

To examine the biological alterations in the peritumoral area, IPA (QIAGEN Redwood City; Content version 52912811, Release Date 2020-06-01) (1) molecular network functional enrichment analysis and (2) core analysis were performed as followings. (1) Nineteen peri-T specific DEGs were uploaded to IPA and connected to the direct interaction of these molecules. The overlay function of the “Diseases and BioFunctions” was performed to identify the “Epilepsy or neurodevelopmental disorder” related molecules. Log2 fold change values of peri-T vs. HC were overlaid with the corresponding molecules. (2) The respective DEG (HC vs. Peri-T, T vs. Peri-T, HC vs. T) lists were uploaded as the input dataset, and then canonical pathway analyses were performed with default settings. As the next step, the comparison analysis was performed by using the results of the canonical pathway analyses. To determine predictive biological alterations specific to the peritumoral area, canonical pathways with activation z-scores between HC and Peri-T and between T and Peri-T greater than 2 (or less than -2) were included, but annotation terms with activation z-scores between HC and T greater than 2 (or less than -2) were excluded.
